# Carbohydrate-Mediated Pregnancy Gut Microbiota and Neonatal Low Birth Weight

**DOI:** 10.3390/nu16091326

**Published:** 2024-04-28

**Authors:** Hong-Ren Yu, Yao-Tsung Yeh, Hong-Tai Tzeng, Hong-Ying Dai, Wei-Chia Lee, Kay L. H. Wu, Julie Y. H. Chan, You-Lin Tain, Chien-Ning Hsu

**Affiliations:** 1 Department of Pediatrics, Kaohsiung Chang Gung Memorial Hospital, Graduate Institute of Clinical Medical Science, Chang Gung University College of Medicine, Kaohsiung 833, Taiwan; 2 Aging and Disease Prevention Research Center, Department of Medical Laboratory Sciences and Biotechnology, Fooyin University, Kaohsiung 831, Taiwan; 3 Institute for Translational Research in Biomedicine, Kaohsiung Chang Gung Memorial Hospital, Chang Gung University College of Medicine, Kaohsiung 833, Taiwan; 4 Department of Urology, Kaohsiung Chang Gung Memorial Hospital, Chang Gung University College of Medicine, Kaohsiung 833, Taiwan; 5 Department of Pediatrics, Kaohsiung Chang Gung Memorial Hospital, Chang Gung University College of Medicine, Kaohsiung 833, Taiwan; 6 Department of Pharmacy, Kaohsiung Chang Gung Memorial Hospital, Kaohsiung 833, Taiwan; 7 School of Pharmacy, Kaohsiung Medical University, Kaohsiung 807, Taiwan

**Keywords:** carbohydrates intake, macronutrients, pregnancy, low birth weight, fetal growth, gut microbiota, maternal health

## Abstract

The effects of gut microbiota on the association between carbohydrate intake during pregnancy and neonatal low birth weight (LBW) were investigated. A prospective cohort study was conducted with 257 singleton-born mother–child pairs in Taiwan, and maternal dietary intake was estimated using a questionnaire, with each macronutrient being classified as low, medium, or high. Maternal fecal samples were collected between 24 and 28 weeks of gestation, and gut microbiota composition and diversity were profiled using 16S rRNA amplicon gene sequencing. Carbohydrates were the major source of total energy (56.61%), followed by fat (27.92%) and protein (15.46%). The rate of infant LBW was 7.8%, which was positively correlated with maternal carbohydrate intake. In the pregnancy gut microbiota, *Bacteroides ovatus* and *Dorea* spp. were indirectly and directly negatively associated with fetal growth, respectively; *Rosenburia faecis* was directly positively associated with neonatal birth weight. Maternal hypertension during pregnancy altered the microbiota features and was associated with poor fetal growth. Microbiota-accessible carbohydrates can modify the composition and function of the pregnancy gut microbiota, thus providing a potential marker to modulate deviations from dietary patterns, particularly in women at risk of hypertension during pregnancy, to prevent neonatal LBW.

## 1. Introduction

Approximately 14.6% of newborns worldwide have low birth weight (LBW), defined as weighing less than 2500 g, amounting to over 20 million infants annually [1]. LBW is closely associated with preterm birth and includes infants who are small for their gestational age (SGA) [1]. More than 80% of neonatal deaths occur in LBW newborns, with two-thirds being preterm and one-third SGA [2]. Notably, LBW is not only a predictor of perinatal death but also increases the risk of adult diseases [3], also known as the developmental origins of health and disease (DOHaD) [4]. In 2012, the World Health Assembly (WHA) endorsed a Comprehensive Implementation Plan on Maternal, Infant, and Young Child Nutrition, setting a target of 30% reduction in LBW live births between 2012 and 2025 [5]. Although there has been some progress in reducing LBW, achieving the global nutritional target requires further effort [1].

Maternal nutrition during pregnancy is a key factor that influences pregnancy and fetal outcomes. Maternal malnutrition can lead to a range of complications for both the mother and the developing fetus, covering LBW, preterm birth, preeclampsia, gestational hypertension, and stillbirth [6]. While nutritional imbalance during pregnancy stands as the primary factor linked to LBW, other factors such as antenatal chemical exposure, maternal illness, stress, and medication usage can also exert influence [7]. Specific dietary patterns, characterized by a high consumption of red and processed meats and fried foods alongside a low intake of vegetables, fruits, whole grains, nuts, and seafood, are associated with LBW [8,9,10]. However, there is currently no conclusive evidence regarding which maternal dietary patterns are beneficial for preventing LBW [8,9,10].

While current dietary guidelines advocate for increased energy intake during pregnancy, specific recommendations regarding the macronutrient composition of the maternal diet are lacking [11]. It is consistently advised to steer clear of simple sugars, processed foods, and trans and saturated fats, as well as to limit the consumption of red and processed meats [12]. Furthermore, any diet that severely restricts any macronutrient class, such as the ketogenic diet due to its carbohydrate deficiency, the Paleo diet because of its exclusion of dairy, and any diet characterized by excessive saturated fats, should be avoided [12,13,14]. Nevertheless, the impact of excessive intake of specific macronutrients during pregnancy on LBW remains uncertain.

During pregnancy, the main sources of carbohydrates in the diet encompass fruits, starchy vegetables, and whole grains. These food groups supply crucial nutrients, including carbohydrates and dietary fiber, which are essential for maternal and fetal health. Nonetheless, excessive intake of sugars, notably from sugar-sweetened beverages, among pregnant women, may pose risks for adverse outcomes and dysbiosis in the gut microbiota. Recent data indicate that heightened sugar intake during pregnancy might correlate with elevated gestational weight gain and the emergence of unfavorable pregnancy and fetal consequences, such as gestational diabetes, preeclampsia, and preterm birth [15]. Despite carbohydrates being a primary energy source for fetuses, a systematic review including 86,461 maternal–child pairs found minimal impact of maternal carbohydrate intake on neonatal birth weight [16]. While some studies reported positive relationships between neonatal birth weight and carbohydrate intake, most studies showed no significant association [16].

The maternal diet during pregnancy has been associated with maternal microbiota, potentially impacting microbial transmission to the neonate and shaping the neonatal microbial profile, which may have health implications [17]. However, the underlying mechanisms remain unknown. While disruptions in the gut microbiome could be linked to maternal high sugar intake, there is insufficient information regarding alterations in the gut microbiome during pregnancy in response to maternal carbohydrate intake and their connection with LBW. Therefore, this study aimed to evaluate the impact of maternal diet on LBW and explore its association with the maternal pregnancy gut microbiome.

## 2. Materials and Methods

### 2.1. Study Design and Participants

This prospective study of pregnant women was conducted between June 2019 and April 2022 at the Medical Center of Kaohsiung Chang Gung Memorial Hospital in Taiwan. We identified women in the second trimester of pregnancy from the Obstetrics and Gynecology clinic for possible enrollment in the study; of these, women who consented to provide fecal samples and complete an intake questionnaire entered the study. The samples used to investigate the associations between maternal diet and fetal outcomes as well as maternal gut microbiota. The data excluded from analysis were multiple gestations, giving birth outside of the study setting, resulting in a lack of fetal outcomes, and stillbirth.

### 2.2. Sample Collection and Comparison Groups

Maternal fecal samples were collected at home between 24 and 28 weeks of gestation (second trimester) using a collection tube containing a DNA Stabilizer. Fecal DNA was extracted from these samples using the QIAmp Fast DNA Stool Mini Kit (Qiagen, Hilden, Germany), following modified instructions from the Aging and Disease Prevention Research Center at Fooyin University.

During the second trimester, pregnant women completed a validated FFQ [18] to report their daily intake of foods and nutrients. FFQ information was analyzed using the Taiwan Nutrients Food Composition Tables [19]. Participants were requested to indicate how many times each food was consumed per month/week/day in the past month. The FFQ includes 37 items in six food categories: whole grains, meat/fish/egg, dairy products, oil/fats/nuts/seeds, vegetables, and fruit. The analysis included assessing the total energy intake (kcal) and the caloric contribution of each macronutrient (carbohydrate, protein, and fat) as a percentage.

To investigate the correlations between maternal diet during pregnancy and fetal outcomes, post hoc comparison analysis first classified each macronutrient intake into three groups: high (>2SD [standard deviations]), medium (within 2SD), and low (<2SD) categories using linear regression analysis adjusted for maternal pregestational body weight. Macronutrient intake >3SD of the average was considered an outlier. Three outliers for carbohydrates, one for protein, and three for fat intake in maternal–child pairs were further excluded from the microbial analyses. The following analyses were compared according to three groups of each macronutrient intake. 

### 2.3. Fetal Outcomes and Maternal Characteristics

The anthropometric and clinical parameters of the mother–child dyads were measured by health professionals and recorded in the electronic medical records (EMR) data system. The primary outcome of interest was fetal outcome, including gestational age in weeks, classification as SGA, appropriate gestational age (AGA), or large gestational age (LGA) based on Taiwanese birth data [20], mode of birth, birth weight, and length at birth were retrieved from the EMR data system.

Maternal health information, including diagnoses of hypertension during pregnancy or preeclampsia, gestational diabetes, anemia, and administration of antibacterial and gastric acid-lowering agents (proton pump inhibitors, H_2_-receptor antagonists, and antacids) during pregnancy, was obtained from the EMR data system. Additional maternal characteristics included pregestational body weight, body mass index (BMI), weight changes from the pregestational stage to late pregnancy (change in BMI, weight gain), and maternal age at birth. 

### 2.4. DNA Extraction and 16S rRNA Amplicon Sequencing

Microbial DNA was extracted from the stool samples (~200 mg) using the QIAamp^®^ DNA Mini Kit (QIAGEN Canada, Mississauga, ON, Canada) following the manufacturer’s protocol. After extraction and purification, the DNA was used as the PCR template for amplification. The 16S rRNA gene was amplified using a specific primer set targeting the bacterial V3–V4 region, and DNA samples were paired-end sequenced (2 × 300 bp) using the Illumina MiSeq platform (Illumina, San Diego, CA, USA) by Majorbio Bio-Pharm Technology Co., Ltd. (Shanghai, China), as previously described [21].

Sequence quality control and construction of a feature table for the sequence data were performed and corrected using QIIME 2 version 2023.2 (https://qiime2.org, assessed on 25 March 2024) [22] and the DADA2 pipeline [23]. The reads were then merged into amplicon sequence variants (ASVs) for downstream analyses.

### 2.5. Statistical Analyses

Alpha diversity, representing the genera richness, was calculated using the Shannon and Chao-1 indices for community diversity and abundance, respectively. The calculation of alpha diversity indices was performed using the “vegan” package in R (https://cran.r-project.org/). Plots were generated using the Python 3.11 packages pandas, seaborn, and matplotlib.

For beta diversity, the Bray–Curtis distance was calculated based on genus-level data using the “vegan” R package. Principal coordinate analysis figures were created using the “ggplot2” R package. Bray–Curtis matrix PERMANOVA was conducted using the Adonis function of the vegan package in R v.4.3.2. The taxonomic compositions of the ASVs were mapped using Greengenes 13_8, with 99% of the OTUs as reference sequences.

The Phylogenetic Investigation of Communities by Reconstruction of Unobserved States (PICRUSt2) pipeline within the QIIME2 plugin was used to predict functional abundance and Kyoto Encyclopedia of Genes and Genomes (KEGG) pathways. Statistical analysis of taxonomic and functional profiles (STAMP) was employed for the statistical tests and visualization of the feature abundance profiles generated by PICRUSt2.

Important pregnancy gut microbiota associated with carbohydrate intake and maternal health status were evaluated using a random forest model, which was based on the proportion of mean changes explained by the variable. Network analyses involving important gut microbiota, KEGG pathways, and maternal characteristics were performed to elucidate the interplay relationship with fetal outcomes using Spearman’s correlation analysis and Cytoscape software (version 3.10.1). The significance level was set at *p* < 0.05. 

## 3. Results

### 3.1. Maternal Macronutrient Pattern during Pregnancy

A total of 257 maternal–child dyads were included in this study. The mean total calorie intake of pregnant women was 1623.87 (±494.08) Kcal, with 56.61% of energy from carbohydrates, 27.92% from protein, and 15.46% from fat. We initially categorized carbohydrate intake into three levels: low (25.59%, n = 65), medium (54.7%, n = 139), and high (19.7%, n = 50). The mean total calories were higher in the high carbohydrate intake group than in the medium- and low carbohydrate intake groups (Table 1). The mean total protein and total fat were higher in the high carbohydrate intake group than in the other groups, but higher proportions of total calories from protein and fat were lower in the medium groups of carbohydrate intake (Table 1).

### 3.2. Pregnancy Carbohydrate Intake and Fetal Outcomes

The characteristics of the pregnant women and fetal outcomes based on carbohydrate intake are summarized in Table 1 (protein and fat intake characteristics are provided in Supplementary Table S1). The rate of LBW was 7.8%, which was higher in pregnant women with a low carbohydrate intake than in those with a medium or high carbohydrate intake (12.31%, 5.76%, and 8%, respectively).

**Figure 1:**
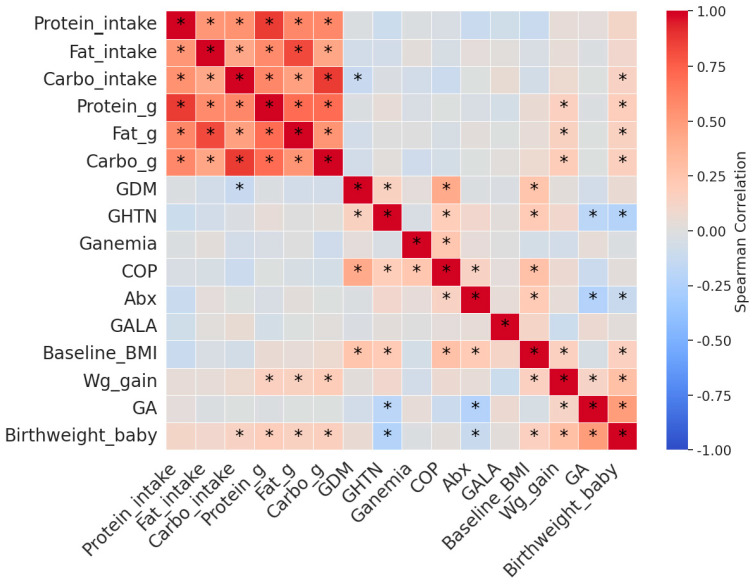
Pairwise correlations among macronutrients during pregnancy, maternal health conditions, and fetal outcomes. * Spearman correlation coefficients with *p* < 0.05 using SciPy library in Python. GDM, gestational diabetes; Ganemia, anemia during pregnancy; GHTN, gestational hypertension/pre-eclampsia; COP, any one of Ganemia, GDM, or GHTN; Abx, anti-bacteria agent; GALA, gastric-acid-lowering agent.

Figure 1 shows the correlations between pregnancy macronutrients, maternal health status, and fetal outcomes. A positive correlation was identified between carbohydrate intake (Carbo_intake: low, medium, and high) and fetal birth weight (birth weight_baby), but there was no correlation with fat or protein intake. In contrast, pregnant women having hypertension during pregnancy or pre-eclampsia (GHTN) showed a negative correlation with fetal birth weight (birthweight_baby).

**Figure 2:**
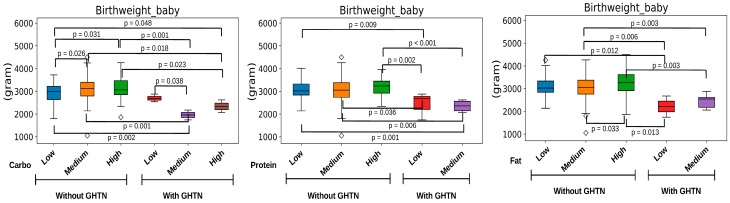
Maternal macronutrient intake during pregnancy and fetal birthweight by pregnant women with or without hypertension/preeclampsia during pregnancy (GHTN). *p*-values were based on Student’s *t*-tests performed between the two groups using the SciPy library in Python. The plots were drawn with the Python packages pandas, seaborn, and matplotlib.

The direction of the correlation between the intake of each macronutrient and fetal birth weight was significantly different according to GHTN (Figure 2). Notably, a correlation between higher carbohydrate intake (carbo) and larger birth weight was not found in pregnant women with GHTN. Other associations with fetal outcomes are shown in Supplemental Figure S1.

### 3.3. Maternal Pregnancy Gut Microbiota and Carbohydrate Intake

**Figure 3:**
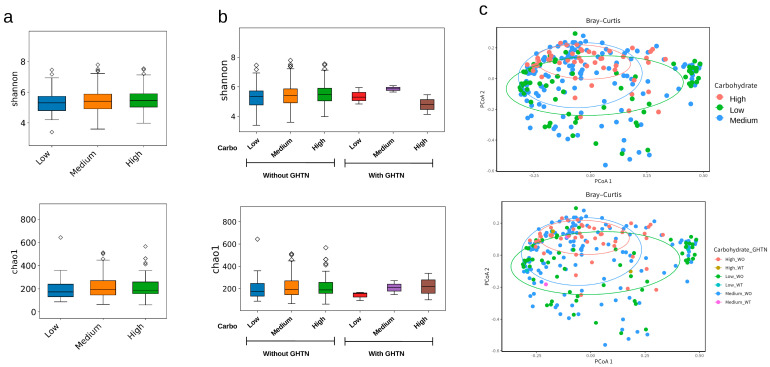
Diversity and carbohydrate-mediated gut microbiota in pregnant women with or without GHTN. (**a**) Alpha diversity represented by Shannon and Chao1 indices in pregnant women. (**b**) Alpha diversity between pregnant women with or without GHTN. (**c**) Beta diversity by carbohydrate intake and maternal GHTN represented by principal coordinate analysis (PCoA).

Carbohydrate intake was not directly associated with alpha diversity as estimated by the Chao1 and Shannon indices (Figure 3a) or with beta diversity (Figure 3c). The alpha diversity (Shannon index) tended to increase with carbohydrate intake. However, this effect was less pronounced in the GHTN group (Figure 3b). Analysis of alpha diversity (Chao1 index) indicated that the richness of the gut microbiota increased with higher carbohydrate intake in both the GHTN and without-GHTN groups, but the extent of the changes in carbohydrate intake differed between the groups (Figure 3b).

**Figure 4:**
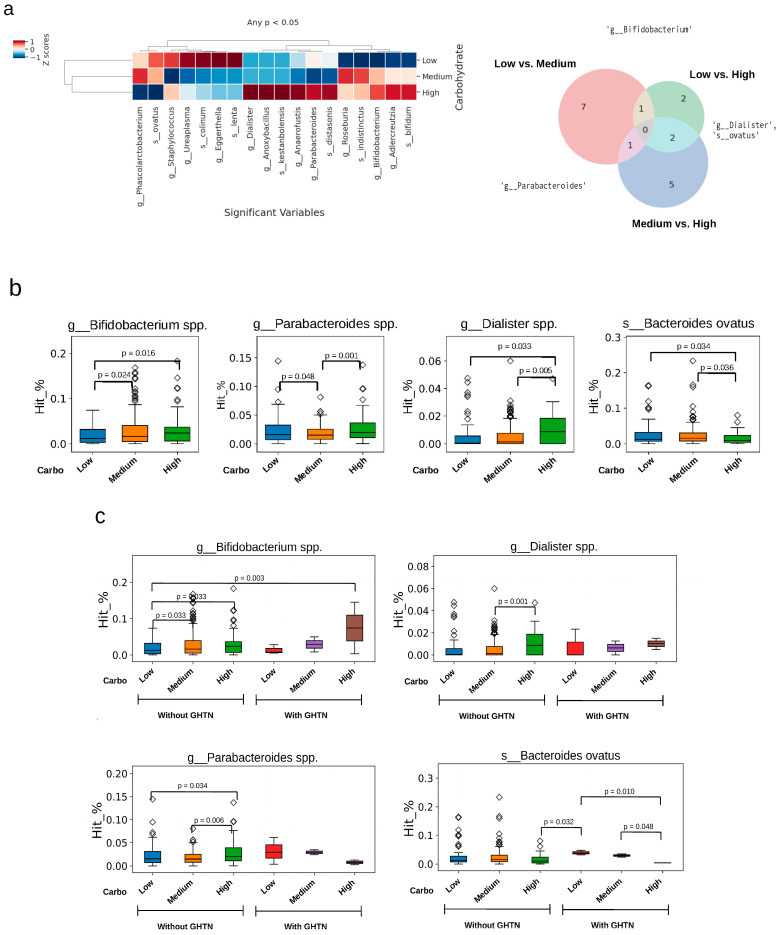
Pregnancy gut microbiota composition’s relative abundance differences by level of carbohydrate intake. (**a**) Compositions of pregnancy gut microbiota; averages and standard deviations were computed using the base function in R 4.3.2. Venn diagrams were drawn with the Venn Diagram package, and heatmaps were generated using the pheatmap package by R 4.3.2. (**b**) Top 4 gut microbiota responded to carbohydrate intake level. (**c**) Direct relationship between carbohydrate intake and gut microbiota altered by GHTN. Student’s *t*-test was used for comparison between the two groups. We calculated the *p*-values using the SciPy library in Python. The plots were drawn with the Python packages pandas, seaborn, and matplotlib.

The abundance of 18 genera and species was affected by carbohydrate intake (Figure 4a). Three bacterial genera and one species were dominant at different levels of carbohydrate intake (e.g., two microbiotas were identified between medium vs. high and low vs. high carbohydrate intake). Greater differences in the abundance of these four gut microbiota compositions according to the level of carbohydrate intake were observed in the *Bacteroides ovatus* (*B. ovatus*) species and the *Bifidobacterium* spp. genus (Figure 4b). The relationship between carbohydrate intake and the relative abundance of *B. ovatus* and *Bifidobacterium* spp. was significantly altered in pregnant women with GHTN (Figure 4c). For instance, the relative abundance of *Bifidobacterium* spp. increased in pregnant women with GHTN, whereas the abundance of *B. ovatus* decreased in pregnant women with GHTN.

### 3.4. Predicted KEGG Pathway and Carbohydrate-Mediated Gut Microbiota

The analysis of predicted KEGG pathways identified four out of 29 pathways: African trypanosomiasis, aminobenzoate degradation, oxidative phosphorylation, and beta-alanine metabolism as most relevant to the variability in carbohydrate intake (Supplemental Figure S2a,b). The potential KEGG pathways involved in carbohydrate intake, African trypanosomiasis, and beta-alanine metabolism differed substantially among pregnant women with GHTN (Supplemental Figure S2c).

### 3.5. Important Maternal Gut Microbiota Compositions Affected by Carbohydrate Nutrition and Maternal Hypertension during Pregnancy

The random forest model confirmed that two genera (*Bifidobacterium* spp. and *Dialister* spp.) and one species (*B. ovatus*) of the pregnancy gut microbiota and two KEGG pathways (aminobenzoate degradation and oxidative phosphorylation) were significantly associated with predicted carbohydrate intake (Supplemental Figure S3a,b).

The most important predictors of maternal GHTN association with carbohydrate intake were one genus (*Dorea* spp.), two species (*Rosenburia feacis and Streptococcus sobrinus*), and a set of six important pathways (cysteine and methionine metabolism, pentose and glucuronate interconversions, other glycan degradation, the phosphotransferase system, nicotinate, nicotinamide metabolism, and amino sugar and nucleotide sugar metabolism) (Supplemental Figure S3c,d).

### 3.6. Key Carbohydrate-Mediated Pregnancy Gut Microbiota and Its Association with Fetal Outcomes

The visualized connection network showed that pregnant women with GHTN had a direct negative association with fetal outcomes (both neonatal birth weight and gestational age), as well as *Dorea* spp. did. Carbohydrates were directly positively associated with neonatal birth weight, and it was directly negatively associated with *B. ovatus* (Supplemental Figure S4a). Correlations among microbial features and functional pathways, fetal outcomes, and macronutrients varied between pregnant women with or without GHTN (Supplemental Figure S4b,c). 

**Figure 5:**
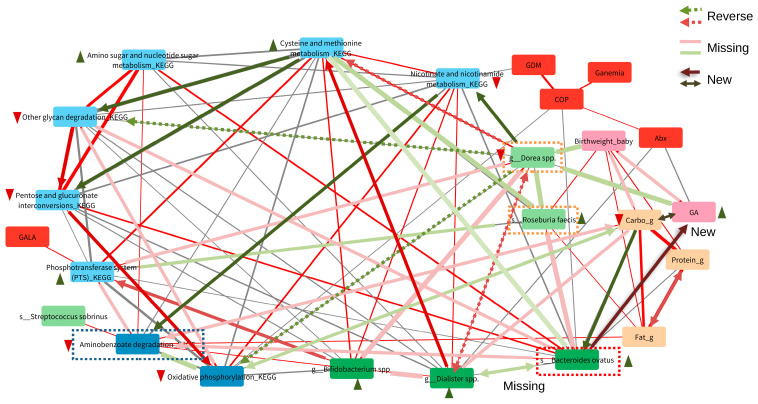
Alterations by maternal GHTN in the network associations among macronutrients, gut microbiota, functional pathways, fetal outcomes, and maternal health. Black line: negative correlation; Red line: positive correlation. Compared to the maternal group without GHTN (pink): alterations (reverse, missing and new correlations) were observed in the maternal GHTN group (green).

**Figure 6:**
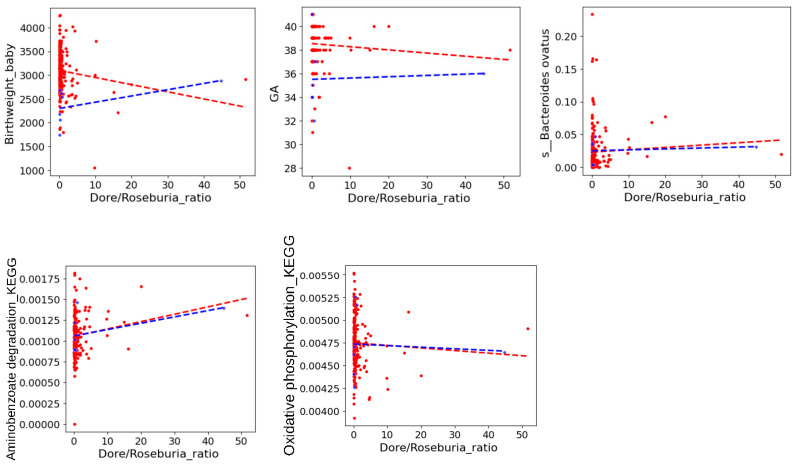
Variability in the *Dorea/Roseburia* ratio between maternal GHTN and without GHTN and its association with fetal outcomes, abundance of *B. ovatus* and corresponding functional pathways. Red line (without GHTN), blue line (GHTN); *p* value was calculated with Spearman correlation coefficients; Only neonatal birthweight was significantly associated with the *Dorea/Roseburia* ratio in the group without GHTN (*p* = 0.024).

The network associations present the direction of the associations altered by maternal GHTN (Figure 5). For example, among pregnant women who developed GHTN, a new direct positive correlation between *B. ovatus* and GA (gestational age), negative correlations between *B. ovatus* and carbohydrates (Carbo_g), and a negative correlation between GA and Carbo_g were observed. In contrast, the negative connection between Dorea spp. and neonatal birth weight, the positive connection between *Roseburia faecis* and neonatal birth weight, and the negative connection between *Roseburia faecis* and *Dorea* spp. disappeared in the group of GHTN. As *Dorea* spp. and *Roseburia faecis* were correlated with *B. ovatus* and highly variable between the maternal groups with and without GHTN, further analyses found that the higher *Dorea/Roseburia faecis (Roseburia)* ratio was associated with lower neonatal birth weight in the maternal group without GHTN (Figure 6). Although the sample size was too small to show the significance in the GHTN group, the reversed trend was found between *Dorea/Roseburia* ratio and neonatal birth weight (Figure 6). These results could explain the observed altered associations by maternal GHTN when examining the interaction effects of macronutrient gut microbiota on fetal outcomes. 

## 4. Discussion

Our research underscores the potential of altering microbiota-accessible carbohydrates to influence the composition and functionality of the gut microbiota throughout pregnancy. This adjustment holds promise as an indicator for adapting dietary practices, particularly for pregnant women at risk of hypertension, with the goal of reducing the likelihood of LBW. In the macronutrients of pregnant women in the study, the primary source of energy was carbohydrates, which is an important contributor to the maternal gut microbiota during pregnancy and fetal growth. Maternal GHTN caused proportional differences in the three macronutrients of the total energy source, which correlated with fetal growth (Figure 2). The top four pregnancy gut microbes reflected a carbohydrate-dominated diet (carbohydrate: fat: protein = 3.8:1.9:1), and three (*B. ovatus*, *Dorea* spp., and *Roseburia faecis*) were sensitive to maternal GHTN in the study population. The taxonomic features of the pregnancy gut microbiota mediated by carbohydrates disrupted by maternal GHTN highlight the importance of host environmental determinants during pregnancy for neonatal outcomes.

Maternal gut microbiota composition and metabolic activity differ before and during pregnancy [24,25]. Numerous studies have suggested associations between the compositions of maternal [26] and/or infant [27,28] gut microbiota and adverse fetal outcomes in many populations. For example, *Ruminococcaceae, Lachnospiraceae,* and *Eubacteriaceae* families identified from maternal fecal samples during pregnancy were important predictors of neonatal birth weight and weight at 1 month; *Ruminococcaceae*, *Lachnospiraceae*, *Eubacteriaceae* families, *Prevotella copri*, and *Slackia isoflavoniconvertents* were associated with gestational age in the rural Zimbabwe cohort [26]. A small sample cohort in Taiwan found that infant gut microbiota compositions and beta diversity were different between SGA and AGA preterm infants at 1 month of age [27]. The study results found that the abundance of specific microbiota composition changes over time (7,14, and 30 days of age). On day 30, a lower abundance of *Klebsiella* (SGA 3.76% vs. AGA 16.05%; *p* = 0.07) and *Enterobacter* (SGA 5.09% vs. AGA 27.25%; *p* = 0.011) was found in SGA infants, implying that SGA influences the gut microbial composition development in early life [27]. Similarly, preterm infant gut microbiota during days 3–4 postpartum was distinct from full-term infant gut microbiota, and spontaneous preterm birth was associated with changes in gut microbiota compositions of both preterm neonates and their mothers’ gut microbiota in the Finland population [28]. 

Host characteristics such as pregnancy weight, gestational weight gain, fasting blood glucose, and place of residence explain 3–5-fold more variance in the gut microbiota composition than the stage of gestation [29]. Previous studies on hypertensive individuals have demonstrated positive correlations between blood pressure and the abundance of *Dialister* and *Parabacteroides* spp. [30]. *Dialister* spp., on the other hand, was found to be significantly depleted in antepartum preeclampsia women compared to uncomplicated pregnant women [31]. Notably, *Dialister* spp. and *Parabacteroides* spp. were less significant in the study cohort. Our findings further suggest that maternal GHTN can alter the carbohydrate-mediated associations between *B. ovatus, Dorea* spp., and poor fetal growth and that between *Roseburia faecis* and positive fetal birth weight.

It is known that high carbohydrate intake was associated with an increased abundance of bacteria involved in carbohydrate metabolism, such as the genera *Bifidobacterium*, *Dialister*, and *Parabacteroides* [32,33]. Few reviews focused on macronutrients or carbohydrates [34,35] in relation to pregnancy and fetal outcomes. Notably, different sources of carbohydrates have variable effects on the glycemic responses (GI) and glycemic load (GL) obtained from different foods [36,37]. Higher total fiber content before and during early pregnancy reduces preeclampsia [38] and gestational diabetes mellitus (GDM) [35], and the increased risk of GDM is correlated with higher GL [39]. The pregnant women in the present study had a lower energy intake (1623.87 ± 494.08 Kcal/day) than in the study of pregnant women conducted in the UK (2329 [1882, 2789] kcal/day) examining carbohydrate intake (302.7 [245.7, 372.9] g/day) [40]. The proportion of energy from carbohydrates was higher in our study cohort (56%) than in Western populations (47.3% to 51%) but similar to a Japanese cohort (55.3%) [16] The proportion of total protein (15.46 ± 2.68%) was within the recommended proportion (25%) [41]. As there were relatively minor effects of protein and fat intake on fetal growth in our study cohort, further research should focus on the sources and quality of carbohydrates and their association with pregnancy gut microbiota compositions and diversity to modulate and improve pregnancy health and birth outcomes. 

The influence of maternal nutrition on gut microbiota modulation is widely accepted. For example, a decrease in alpha- diversity of pregnancy gut microbiota in 20–26 weeks of gestation was associated with spontaneous preterm birth, especially in the class of *Betaproteobacteria*, and was attributable to pregnant women with a low-fiber, high-fat diet in the multi-ethnic cohort in the USA [42]. However, the preferred healthy dietary pattern for preventing adverse maternal and fetal outcomes remains uncertain. Because each microbiota determines which carbohydrate is metabolized, the availability of microbiota-accessible carbohydrates (MAC) determines the composition and function of the gut microbiota and supports host homeostasis [43]. The intake of different MACs is associated with the proliferation of different microorganisms. For example, resistant starch is associated with *Ruminococcus*, *Eubacterium rectale*, and *Roseburia*, whereas fructans, polydextrose, fructooligo-saccharides, and galactooligo saccharides are associated with intestinal Bifidobacteria and *Lactobacilli* proliferation [44].

Although *Bacteroides* is a key bacterium involved in carbohydrate metabolism [32,45], our findings suggest that high carbohydrate intake is linked to a reduced abundance of *B. ovatus*, particularly in the GHTN group. Considering that *B. ovatus* is more abundant in women with hypertension compared to normotensive women [46], our data imply that disruptions in *B. ovatus* in response to carbohydrate intake might be associated with hypertension during pregnancy. Short-chain fatty acids are metabolites of MAC produced in the gut microbiota and may serve as intermediates in various pathways [47]. Further research is needed to confirm the mechanisms underlying the connection between key gut microbiota diversity (e.g., *B. ovatus*, *Roseburia faecis*, and *Dorea* spp.) and different carbohydrates, and their roles in response to maternal BP regulation during pregnancy.

Our analysis has some limitations. In this study, we explicitly reported epidemiological associations between maternal macronutrients, pregnancy gut microbiota, and fetal birth weight, and our selected cohort may not completely represent the entire population of pregnant women. This prospective cohort study is the first observational maternal–child cohort study, and additional studies from independent cohorts are needed to strengthen our findings. Although we reported a potential association between carbohydrate-mediated pregnancy gut microbiota and neonatal birth weight, future studies are needed to elucidate the temporal relationship between dietary patterns during specific pregnancy period and gut microbiota. Differences in gut microbiota communities may vary across different stages of gestation [48], and individual dietary habits may not be fully represented in a single stool sample collected within a specific time frame. The FFQ was limited to considering the consumption of supplement uses of probiotics, prebiotics, or yogurt that could modulate gut microbiota compositions. 

## 5. Conclusions

Our analyses illustrate that the pregnancy gut microbiota, primarily its carbohydrate-mediated microbiota features, is an important contributor to neonatal birth weight. We found that maternal hypertension during pregnancy altered the microbiota features and corresponding functional pathways, resulting in adverse fetal growth. The findings of this study will shape directions for future research and the development of dietary interventions to prevent poor pregnancy and fetal growth.

## Figures and Tables

**Table 1:** Maternal nutrition, characteristics, and fetal outcomes among all and the three carbohydrate intake groups.

	All (n = 257)		Carbohydrate Intake ^1^						*p-*Value
			Low (n = 65)		Medium (n = 139)		High (n = 50)		
**Maternal diet during pregnancy**									
Total calories intake (Kcal)	1623.87	(494.08)	1156.44	(245.06)	1583.47	(265.27)	2237.37	(352.80)	<0.0001
Protein (%)	15.46	(2.68)	16.58	(3.01)	15.42	(2.56)	14.27	(1.96)	0.0069
Fat (%)	27.92	(6.34)	29.45	(6.78)	27.80	(5.68)	26.13	(7.14)	<0.0001
Carbohydrate (%)	56.61	(6.89)	53.96	(7.27)	56.78	(6.33)	59.60	(6.85)	<0.0001
Total protein (g)									
Mean (SD)	62.77	19.62	48.42	(13.26)	62.17	(17.36)	80.11	(14.00)	<0.0001
Mmedian (25h, 75th)	60.40	(49.10, 75.50)	48.50	(37.80, 55.30)	59.60	(49.90, 69.90)	78.75	(69.90, 88.30)	<0.0001
Total fat (g)									
Mean (SD)	51.59	23.02	39.37	(15.51)	50.18	(16.07)	67.51	(31.34)	<0.0001
Median (25h, 75th)	48.40	(36.40, 62.20)	36.90	(28.60, 46.90)	48.60	(39.60, 60.90)	64.00	(48.50, 78.50)	<0.0001
Total Carbohydrate (g)									
Mean (SD)	230.47	71.78	155.00	(26.39)	224.02	(31.46)	331.45	(35.64)	<0.0001
Median (25h, 75th)	220.00	(178.50, 263.90)	157.00	(139.10, 171.50)	226.60	(196.20, 250.40)	325.80	(306.50, 353.20)	<0.0001
Fibers (mg), mean (SD)	13.25	(5.19)	10.57	(4.13)	12.92	(4.36)	16.92	(5.49)	<0.0001
**Fetal outcomes**									
Male infant, n (%)	126	(49.03)	33	(50.77)	66	(47.48)	25	(50.00)	0.8930
Birth weight (Kg), n (%)									
<2.5	20	(7.78)	8	(12.31)	8	(5.76)	4	(8.00)	0.0747
2.5 to <4	217	(84.44)	56	(86.15)	119	(85.61)	39	(78.00)	.
≥4	20	(7.78)	1	(1.54)	12	(8.63)	7	(14.00)	.
Gestational age category, n (%)									
SGA	27	(10.51)	11	(16.92)	13	(9.35)	3	(6.00)	0.0702
AGA	190	(73.93)	49	(75.38)	103	(74.10)	35	(70.00)	.
LGA	40	(15.56)	5	(7.69)	23	(16.55)	12	(24.00)	.
Birth mode, n (%)									
Vaginal	175	(68.09)	44	(67.69)	98	(70.50)	31	(62.00)	0.5403
Caesarean	82	(31.91)	21	(32.31)	41	(29.50)	19	(38.00)	.
**Maternal Health**									
Change in BMI, n (%) ^2^									
<25th	58	(22.57)	19	(29.23)	30	(21.58)	7	(14.00)	0.2620
25th to <50th	61	(23.74)	19	(29.23)	32	(23.02)	10	(20.00)	.
50th to <75th	66	(25.68)	12	(18.46)	37	(26.62)	17	(34.00)	.
≥75th	72	(28.02)	15	(23.08)	40	(28.78)	16	(32.00)	.
Weight gain range by recommendation, n (%) ^2^									
Below	96	(37.35)	29	(44.62)	51	(36.69)	15	(30.00)	0.5506
Within	96	(37.35)	20	(30.77)	53	(38.13)	22	(44.00)	.
Above	65	(25.29)	16	(24.62)	35	(25.18)	13	(26.00)	.
Comorbid conditions during the pregnancy, n (%)									
Diabetes	33	(12.84)	12	(18.46)	18	(12.95)	3	(6.00)	0.1435
Hypertension/Pre-eclampsia	7	(2.72)	3	(4.62)	2	(1.44)	2	(4.00)	0.3629
Anemia	12	(4.67)	5	(7.69)	5	(3.60)	2	(4.00)	0.4226
Other gestational comorbidities	110	(38.91)	29	(44.62)	52	(37.41)	19	(38.00)	0.6028
Any one of the above	118	(45.91)	35	(53.85)	62	(44.60)	21	(42.00)	0.3646
Medication uses during the pregnancy, n (%)									
Antibacterial	107	(41.63)	28	(43.08)	56	(40.29)	22	(44.00)	0.8723
Gastric-acid-lowering ^3^	62	(24.12)	13	(20.00)	35	(25.18)	13	(26.00)	0.6752

^1^ Carbohydrate intake was classified by the regression mean carbohydrate intake (g) and standard deviation (SD) adjusted by mother’s pre-gestation body weight as: low (≤−2SD), medium (±2SD), and high (2SD to 3SD); Three outliers (>3SD) for carbohydrates intake in maternal–child pairs were further excluded from the microbial analyses (n = 254). ^2^ Changes in body mass index (BMI) were classified based on the change between late pregnancy and pre-gestational period; weight gain range was categorized based on the weight change by 2009 Institute of Medicine recommendations (https://www.acog.org/clinical/clinical-guidance/committee-opinion/articles/2013/01/weight-gain-during-pregnancy). ^3^ Gastric-acid-lowering agents (proton pump inhibitors, H2-receptor antagonists, and anti-acids). *p* value < 0.05, it refers to a statistical difference in the mean value between multiple groups (ANOVA analyses) or number of patients between multiple groups (Chi-squared or Fisher’s exact tests).

## Data Availability

The data used in this study are available from the corresponding authors after reasonable request due to privacy and ethical restrictions.

## Supplementary Materials

The following supporting information can be downloaded at: https://www.mdpi.com/article/10.3390/nu16091326/s1. Figure S1: Factors associated with fetal growth by carbohydrate intake and maternal health conditions during pregnancy; (a) gestational age; (b) neonatal birth weight. Figure S2: Kyoto Encyclopedia of Genes and Genomes (KEGG) pathways associated with carbohydrate intake. (a) The top 29 most important predicted pathways and four that were mainly related to carbohydrate intake. (b) Predicted KEGG pathways involved in carbohydrate intake. (c) GHTN-altered carbohydrate-mediated predicted KEGG pathways. Figure S3: Important variables associated with random forest-predicted carbohydrate intake and maternal GHTN. (a) Top 30 important gut microbiota and three microbiotas associated with predicted carbohydrate intake; (b) top 30 important predicted KEGG pathways and two pathways associated with predicted carbohydrate intake; (c) top 30 important gut microbiota and three microbiotas overlapping with predicted GHTN; (d) top 30 important gut microbiota and six predicted KEGG pathways overlapping with predicted GHTN. Figure S4: Network analysis. (a) All pregnant women; (b) pregnant women without GHTN; (c) pregnant women with GHTN. Table S1: Maternal nutrition, characteristics, and fetal outcomes according to total protein and fat intake categories.
