# Supplementary material for: Carbohydrate-Mediated Pregnancy Gut Microbiota and Neonatal Low Birth Weight
Source: Nutrients. 2024 Apr 28;16(9):1326. doi: 10.3390/nu16091326 (PMC11085476; doi:10.3390/nu16091326)
Supplement: Supplementary file 1 [file nutrients-16-01326-s001.zip › nutrients-2959409-supplementary.pdf]

Carbohydrate-mediated pregnancy gut microbiota and neonatal low birth weight

Supplementary material

Supplemental Figure S1: Factors associated with fetal growth by carbohydrate intake and maternal health conditions during pregnancy; (a) gestational age; (b) neonatal birth weight.

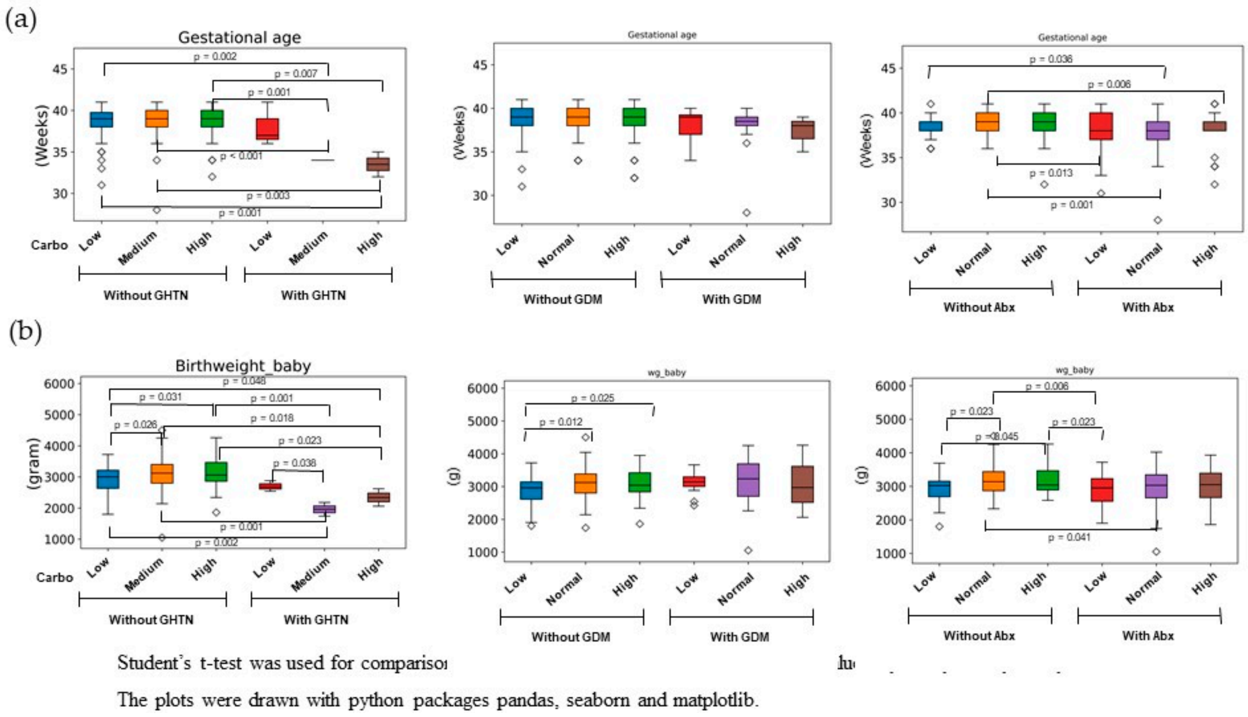

Student’s t-test was used to compare two groups. The P-values were calculated using the SciPy library in Python. The plots were drawn using the Python packages pandas, seaborn, and matplotlib.

Supplemental Figure S2. Kyoto Encyclopedia of Genes and Genomes (KEGG) pathways associated with carbohydrate intake. (a) The top 29 most important predicted pathways and four that were mainly related to carbohydrate intake; (b) Predicted KEGG pathways involved in carbohydrate intake; (c) GHTN altered carbohydrate-mediated predicted KEGG pathways.

(a)

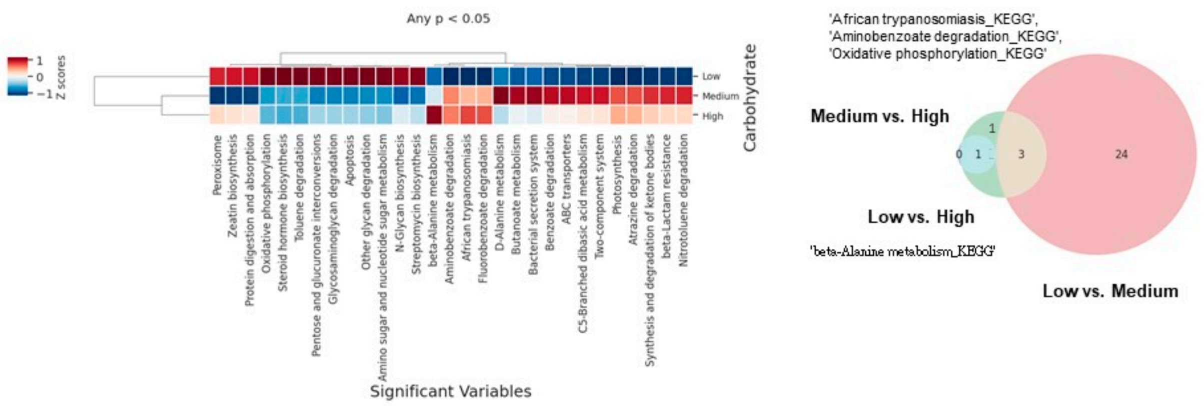

(b)

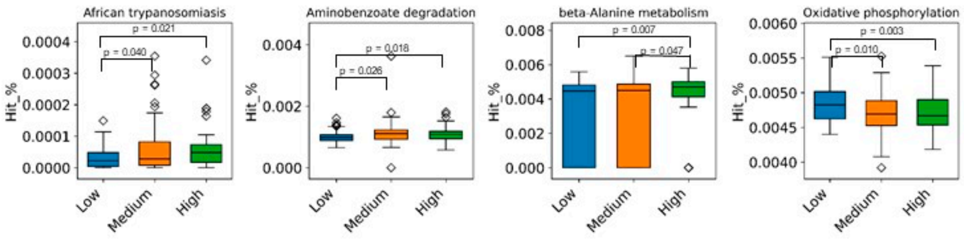

Student's t-test was used for comparison between two groups. We calculated the p-values by SciPy library in Python. The plots were drawn with python packages pandas, seaborn and matplotlib.

(c)

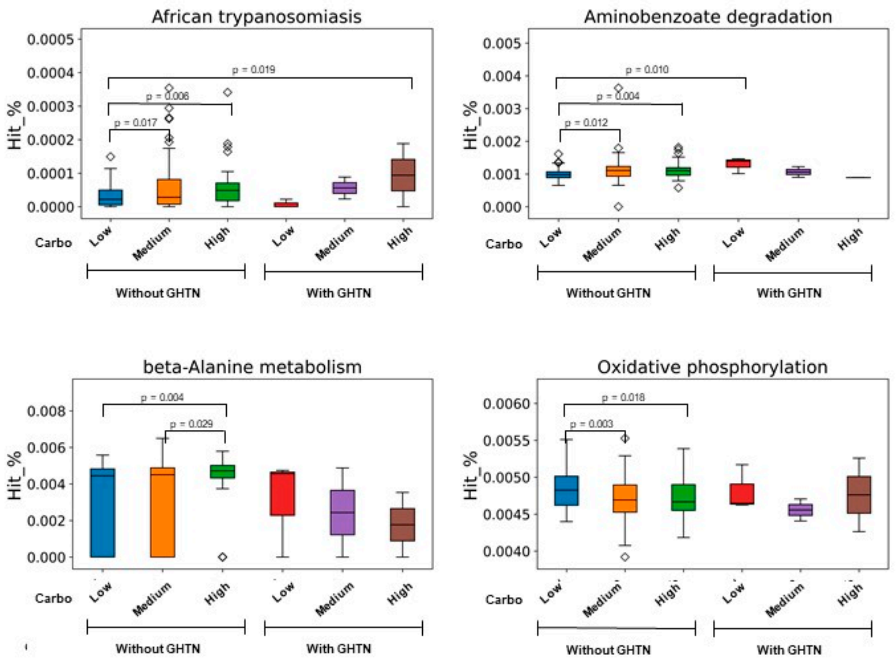

Supplemental Figure S3. Important variables associated with random forest-predicted carbohydrate intake and maternal GHTN.

a. Top 30 most important gut microbiota and three microbiotas associated with predicted carbohydrate intake.

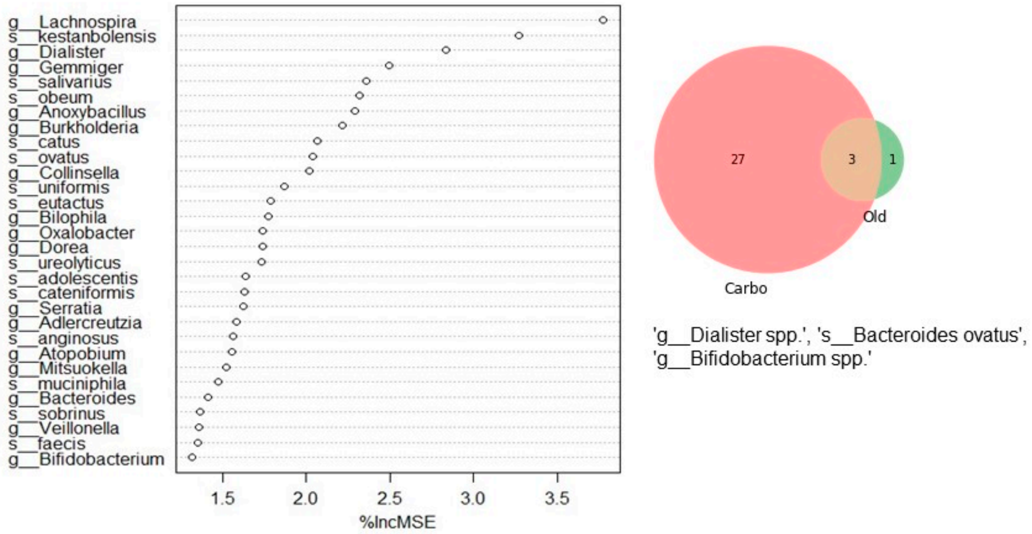

b. Top 30 important predicted KEGG pathways and two pathways associated with carbohydrate intake.

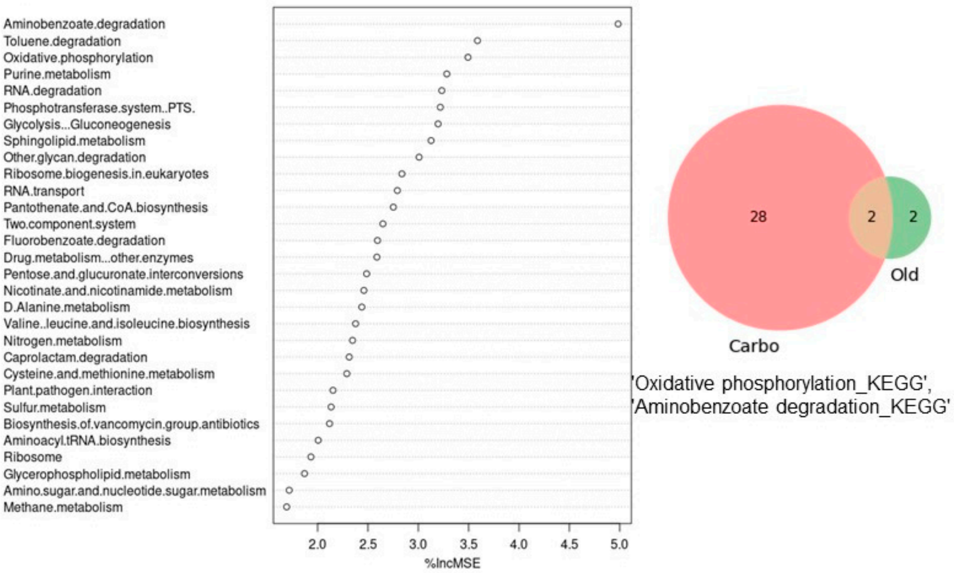

c. Top 30 important gut microbiota and three microbiotas overlapping with predicted GHTN.

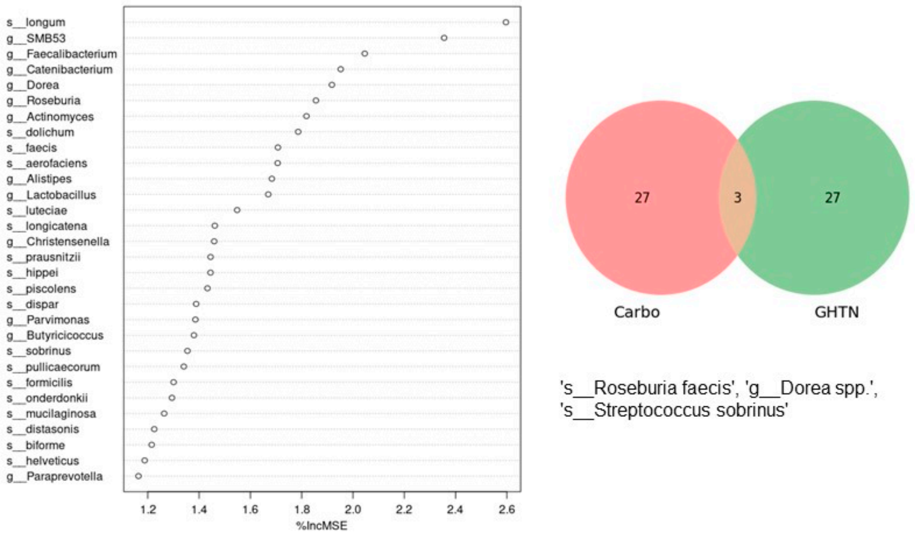

d. Top 30 important gut microbiota and six predicted KEGG pathways overlapping with predicted GHTN.

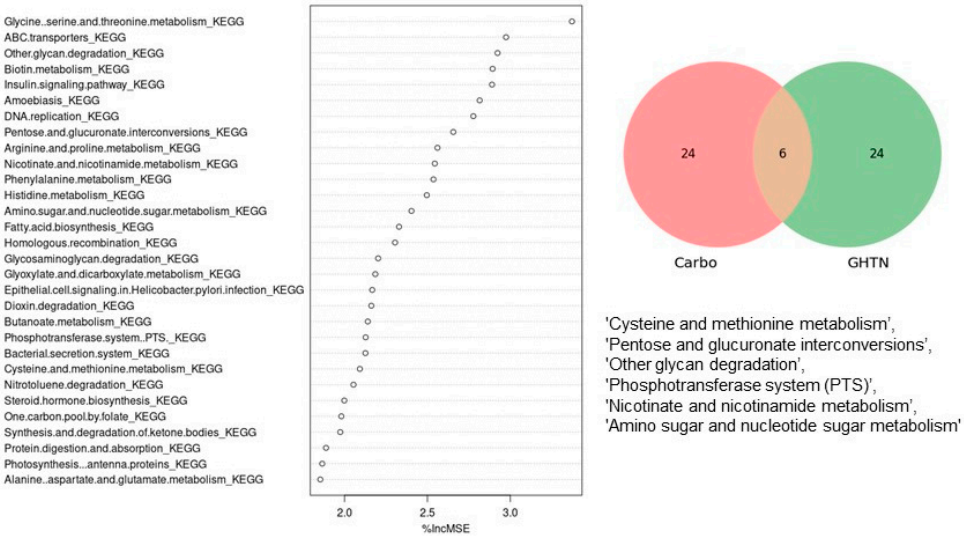

Supplemental Figure S4. Network analysis.

a. All pregnant women

The associations among the six identified pregnancy gut microbiota, eight predicted KEGG pathways of interest, and three macronutrients were combined with six maternal health conditions to assess the connection with fetal outcomes in network analyses (red line: positive correction; grey line: negative correlation).

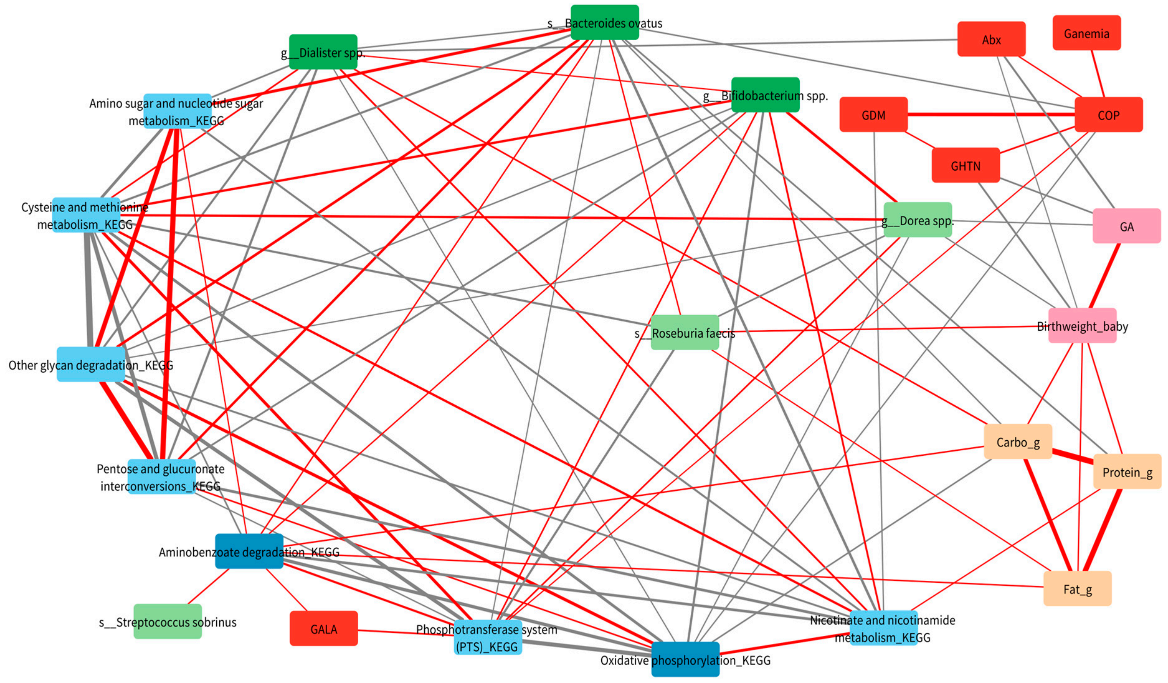

b. Pregnant women without GHTN

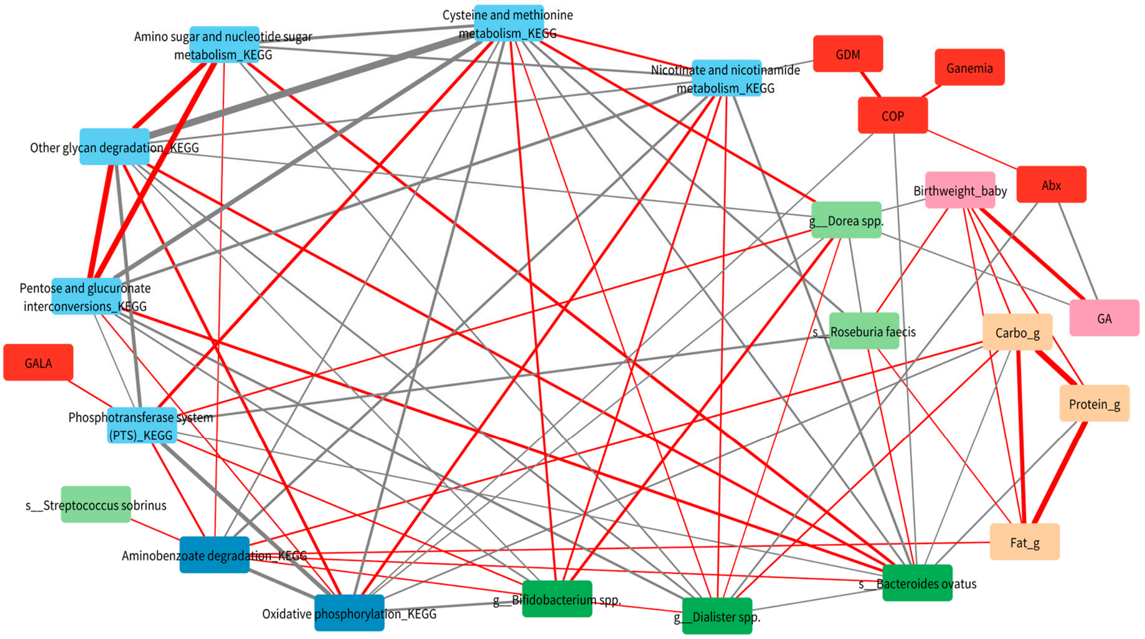

c. Pregnant women with GHTN.

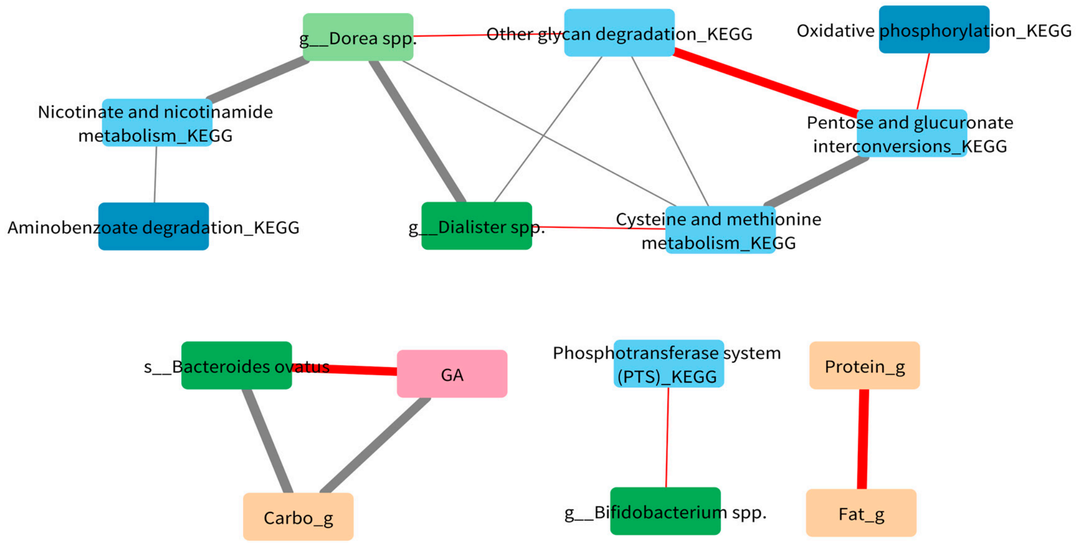

Table S1. Maternal nutrition, characteristics, and fetal outcomes according to total protein and fat intake categories

Characteristics	Protein intake (n=256)			Fat intake (n=254)		
	Low (n=64)	Medium (n=142)	High (n=50)	Low (n=52)	Medium (n=169)	High (n=33)
**Maternal diet during pregnancy**						
Total calories intake（Kcal）	1178.78 (257.50)	1614.26 (338.79)	2196.92 (486.24)	1147.08 (260.35)	1623.66 (340.74)	2195.43 (396.25)
Protein (%)	13.98 (2.13)	15.56 (2.41)	17.07 (3.06)	15.44 (2.58)	15.72 (2.71)	14.55 (2.30)
Fat (%)	26.03 (6.58)	28.61 (6.03)	28.24 (6.46)	21.74 (3.82)	28.33 (5.11)	34.45 (5.30)
**Carbohydrate (%)**	**60.00 (6.44)**	**55.84 (6.71)**	**54.69 (6.52)**	**62.82 (5.37)**	**55.95 (6.02)**	**51.01 (5.68)**
**Infant**						
Male infant, n (%)	31 (48.44)	75 (52.82)	**25 (50.00)**	27 (51.92)	91 (53.85)	**11 (33.33)**
**Birthweight (Kg), mean (SD)**						
**<2.5**	**6 (9.38)**	**12 (8.45)**	**2 (4.00)**	**5 (9.62)**	**12 (7.10)**	**3 (9.09)**
2.5 to <4	56 (87.50)	117 (82.39)	43 (86.00)	42 (80.77)	149 (88.17)	23 (69.70)
≥4	2 (3.13)	13 (9.15)	5 (10.00)	5 (9.62)	8 (4.73)	7 (21.21)
**Gestational age (week), n (%)**						
28-36	6 (9.38)	18 (12.68)	3 (6.00)	3 (5.77)	20 (11.83)	4 (12.12)
37-39	43 (67.19)	89 (62.68)	31 (62.00)	35 (67.31)	107 (63.31)	19 (57.58)
**40-41**	**15 (23.44)**	**35 (24.65)**	**16 (32.00)**	**14 (26.92)**	**42 (24.85)**	**10 (30.30)**
**Gestational age category, n (%)**						
**SGA (<10th birthweight)**	**6 (9.38)**	**18 (12.68)**	**3 (6.00)**	**3 (5.77)**	**20 (11.83)**	**4 (12.12)**
AGA (10th -90th birthweight)	43 (67.19)	89 (62.68)	31 (62.00)	35 (67.31)	107 (63.31)	19 (57.58)
LGA (>90th birthweight)	15 (23.44)	35 (24.65)	16 (32.00)	14 (26.92)	42 (24.85)	10 (30.30)
**Birth mode, n (%)**						
Vaginal	41 (64.06)	97 (68.31)	36 (72.00)	33 (63.46)	118 (69.82)	22 (66.67)
Caesarean	23 (35.94)	45 (31.69)	14 (28.00)	19 (36.54)	51 (30.18)	11 (33.33)
**Maternal characteristics**						

**Changes in BMI (percentile)[2], n (%)**						
<25th	15 (23.44)	36 (25.35)	7 (14.00)	14 (26.92)	34 (20.12)	9 (27.27)
25th to <50th	17 (26.56)	36 (25.35)	8 (16.00)	13 (25.00)	39 (23.08)	9 (27.27)
50th to <75th	15 (23.44)	34 (23.94)	17 (34.00)	12 (23.08)	49 (28.99)	5 (15.15)
≥75th	17 (26.56)	36 (25.35)	18 (36.00)	13 (25.00)	47 (27.81)	10 (30.30)
**Gestational morbidity during the pregnancy, n (%)**						
Diabetes	6 (9.38)	24 (16.90)	3 (6.00)	8 (15.38)	23 (13.61)	2 (6.06)
Hypertension/Pre-eclampsia	3 (4.69)	4 (2.82)	0 (0.00)	2 (3.85)	5 (2.96)	0 (0.00)
Anemia	4 (6.25)	5 (3.52)	3 (6.00)	3 (5.77)	6 (3.55)	3 (9.09)
Other comorbid conditions	32 (50.00)	63 (44.37)	23 (46.00)	28 (53.85)	72 (42.60)	18 (54.55)
**Medication uses during the pregnancy, n (%)**						
Antibacterial	35 (54.69)	52 (36.62)	19 (38.00)	24 (46.15)	63 (37.28)	19 (57.58)
Gastric acid lowering[3]	18 (28.13)	35 (24.65)	9 (18.00)	14 (26.92)	37 (21.89)	10 (30.30)

1. Protein intake was classified by the regression mean total protein intake (g) and standard deviation (SD) adjusted by mother's pre-gestation body weight as: low (<=-2SD), medium (±2SD), and high (2SD to 3SD; Fat intake groups applied the same SD criteria.
2. Changes in BMI (body mass index) were classified based on the change between late pregnancy and pre-gestational period.
3. Gastric acid lowering agents (proton pump inhibitors, H2-receptor antagonists, and anti-acids)
